# Correction: Variable Expression of PIK3R3 and PTEN in Ewing Sarcoma Impacts Oncogenic Phenotypes

**DOI:** 10.1371/journal.pone.0120830

**Published:** 2015-03-20

**Authors:** The PLOS ONE Staff

## Notice of Republication

This article was republished on February 9, 2015, to remove a paragraph in the Results section, which was an erroneous duplication of the Figure 6 legend. The publisher apologizes for the error. Please download this article again to view the correct version. The originally published, uncorrected article and the republished, corrected article are provided here for reference.

## Supporting Information

S1 File Originally published, uncorrected article. (PDF)

S2 File Republished, corrected article. (PDF)
