# Supplementary figures and images for: Variable Expression of PIK3R3 and PTEN in Ewing Sarcoma Impacts Oncogenic Phenotypes
Source: PLoS One. 2015 Jan 20;10(1):e0116895. doi: 10.1371/journal.pone.0116895 (PMC4300218; doi:10.1371/journal.pone.0116895)

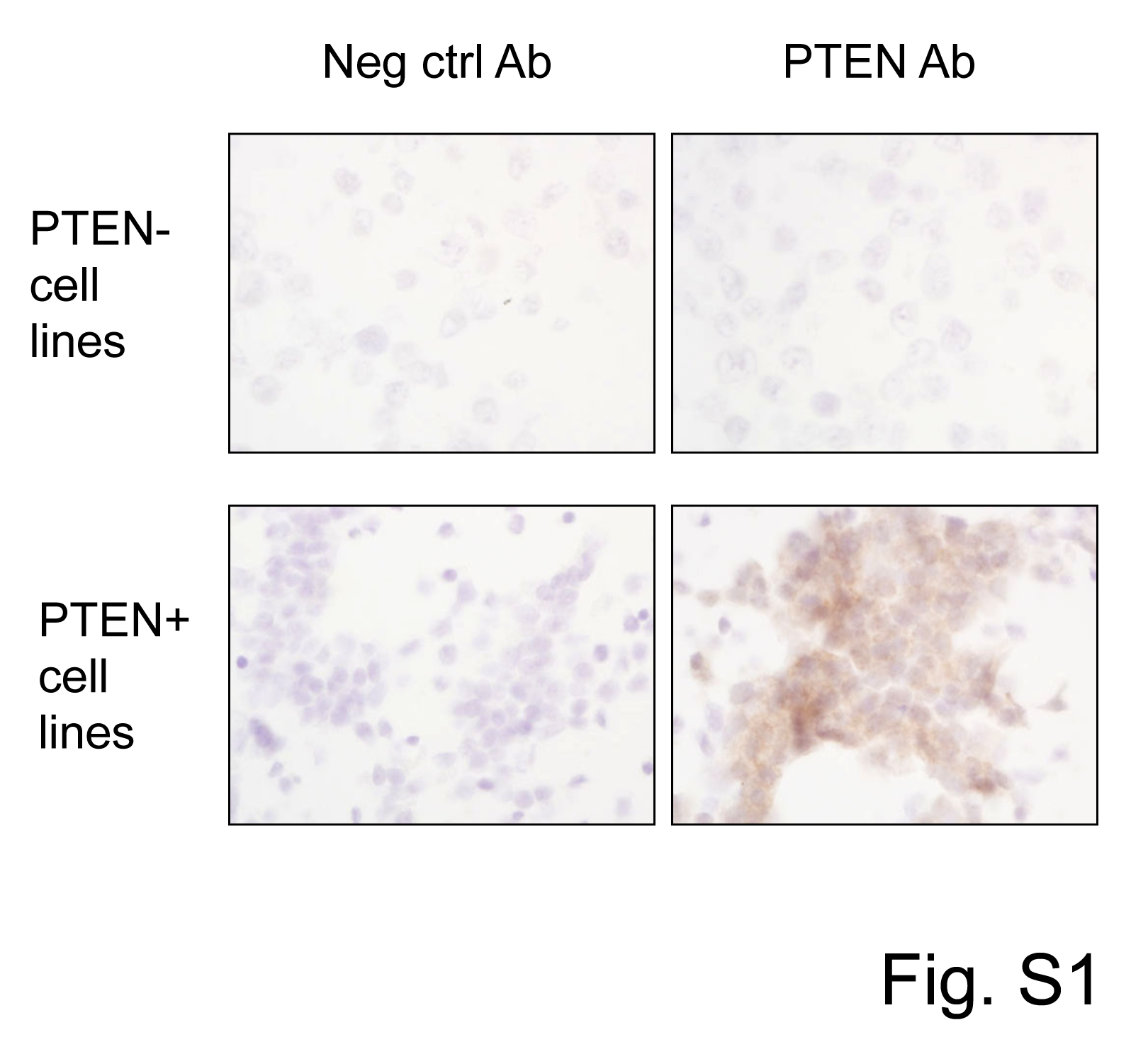

Supplement: S1 Fig — Specificity of PTEN immunohistochemical staining protocol was verified using formalin-fixed, paraffin-embedded pellets of PTEN-positive and PTEN-negative Ewing Sarcoma cell lines (as determined in Fig. 3). See Methods for protocol details. (TIF) [file pone.0116895.s001.tif]

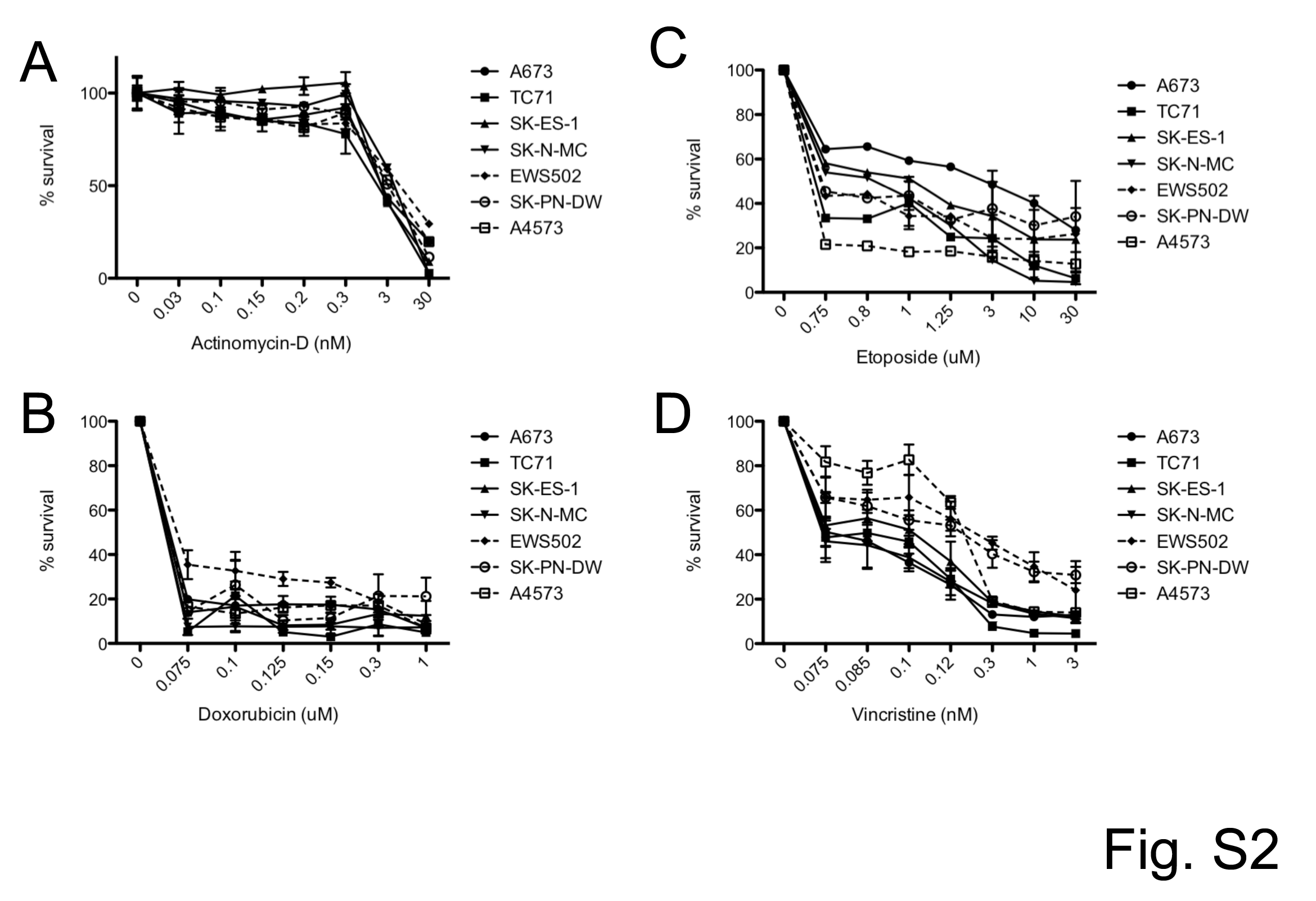

Supplement: S2 Fig — (A-D) Cell survival upon treatment with the indicated agents was compared between PTEN-positive (solid lines) and PTEN-negative (dashed lines) using an MTT assay, as described in Methods. (TIF) [file pone.0116895.s002.tif]
