# Supplementary material for: Correction: Variable Expression of PIK3R3 and PTEN in Ewing Sarcoma Impacts Oncogenic Phenotypes
Source: PLoS One. 2015 Mar 20;10(3):e0120830. doi: 10.1371/journal.pone.0120830 (PMC4368794; doi:10.1371/journal.pone.0120830)
Supplement: S1 File — (PDF) [file pone.0120830.s001.pdf]

PLOS | ONE

RESEARCH ARTICLE

Variable Expression of PIK3R3 and PTEN in Ewing Sarcoma Impacts Oncogenic Phenotypes

Brian F. Niemeyer[‡], **Janet K. Parrish**[‡], **Nicole S. Spoelstra, Teresa Joyal, Jennifer K. Richer, Paul Jedlicka***

Department of Pathology, University of Colorado Denver, Anschutz Medical Campus, Aurora, CO, United States of America

*paul.jedlicka@ucdenver.edu

‡ These authors contributed equally to this work.

CrossMark
click for updates

OPEN ACCESS

Citation: Niemeyer BF, Parrish JK, Spoelstra NS, Joyal T, Richer JK, et al. (2015) Variable Expression of PIK3R3 and PTEN in Ewing Sarcoma Impacts Oncogenic Phenotypes. PLoS ONE 10(1): e116895. doi:10.1371/journal.pone.0116895

Editor: Irina U. Agoulnik, Florida International University, United States of America

Received: May 2, 2014

Accepted: December 16, 2014

Published: January 7, 2015

Copyright: © 2015 Niemeyer et al. This is an open-access article distributed under the terms of the Creative Commons Attribution License, which permits unrestricted use, distribution, and reproduction in any medium, provided the original author and source are credited.

Data Availability: The authors confirm that all data underlying the findings are fully available without restriction. All relevant data are within the paper and its Supporting Information files.

Funding: Funding from Boettcher Foundation's Webb-Waring Biomedical Research Program. The funders had no role in study design, data collection and analysis, decision to publish, or preparation of the manuscript.

Competing Interests: The authors have declared that no competing interests exist.

Abstract

Ewing Sarcoma is an aggressive malignancy of bone and soft tissue affecting children and young adults. Ewing Sarcoma is driven by EWS/Ets fusion oncoproteins, which cause widespread alterations in gene expression in the cell. Dysregulation of receptor tyrosine kinase signaling, particularly involving IGF-1R, also plays an important role in Ewing Sarcoma pathogenesis. However, the basis of this dysregulation, including the relative contribution of EWS/Ets-dependent and independent mechanisms, is not well understood. In the present study, we identify variable expression of two modifiers of PI3K signaling activity, PIK3R3 and PTEN, in Ewing Sarcoma, and examine the consequences of this on PI3K pathway regulation and oncogenic phenotypes. Our findings indicate that PIK3R3 plays a growth-promotional role in Ewing Sarcoma, but suggest that this role is not strictly dependent on regulation of PI3K pathway activity. We further show that expression of PTEN, a well-established, potent tumor suppressor, is lost in a subset of Ewing Sarcomas, and that this loss strongly correlates with high baseline PI3K pathway activity in cell lines. In support of functional importance of PTEN loss in Ewing Sarcoma, we show that re-introduction of PTEN into two different PTEN-negative Ewing Sarcoma cell lines results in downregulation of PI3K pathway activity, and sensitization to the IGF-1R small molecule inhibitor OSI-906. Our findings also suggest that PTEN levels may contribute to sensitivity of Ewing Sarcoma cells to the microtubule inhibitor vincristine, a relevant chemotherapeutic agent in this cancer. Our studies thus identify PIK3R3 and PTEN as modifiers of oncogenic phenotypes in Ewing Sarcoma, with potential clinical implications.

Introduction

Ewing Sarcoma is the second most common bone and soft tissue malignancy in children and young adults [1]. Ewing Sarcoma is driven by recurrent EWS/Ets oncogenic fusions, which, through gain-of-function transcriptional activity, and possibly other mechanisms, result in dysregulation of expression of many genes, as well as non-coding RNAs [2–5]. As in all cancers, the ultimate outcome of oncogene-driven dysregulation of gene expression is promotion of the malignant phenotype. However, as also true in all cancers, the precise mechanistic connections between oncogene-driven gene expression changes and phenotypic output, as well as the identity of key gene expression changes essential to specific malignant phenotypes, are less well understood. It is also now appreciated that some Ewing Sarcomas manifest genetic alterations other than an EWS/Ets oncogenic fusion, for example loss of function of p53 and the p16INK4/p14ARF locus [6]. However, the occurrence and frequency of other such alterations, and their potential contribution to the Ewing Sarcoma malignant phenotype, largely remain to be characterized.

A variety of growth factor signaling pathways are known to be dysregulated in Ewing Sarcoma [2, 3]. The IGF signaling pathway is perhaps the most extensively studied and, to date, the chief pathway pharmacologically drugged in this cancer [7]. Other important growth factor signaling pathways upregulated in Ewing Sarcoma include members of the EGF [8], FGF [9] and Ephrin [10] families, which share downstream molecular machinery, namely MAPK and PI3K signaling cascades, with the IGF pathway. There is evidence that EWS/Ets fusion-driven mechanisms contribute to the upregulation of growth factor signaling in Ewing Sarcoma [11–13]. However, the frequently observed wide range of pathway activity among Ewing Sarcoma cell lines and tumors with the same oncogenic driver fusions suggests that EWS/Ets-independent mechanisms also play a role [8–10]. EWS/Ets-independent mechanisms of signaling pathway activation, in particular, could play an important role in differential biologic behavior and therapy response in Ewing Sarcoma, as recently demonstrated for a member of the EGF family [8].

The PI3K signaling pathway has been shown to play a particularly important role in phenotypes relevant to the aggressive biologic behavior of Ewing Sarcoma [14, 15]. Interestingly, PI3K pathway activity in different Ewing Sarcoma cells lines is quite variable, suggesting an EWS/Ets-independent component of regulation [16]. Activation of PI3K pathway signaling, through loss of the pathway inhibitor PTEN and other mechanisms, is a common and well-characterized pro-oncogenic mechanism in non-fusion driven cancers. Whether similar mechanisms are at play in Ewing Sarcoma largely remains to be defined. In the present manuscript, we examine the expression and role of two regulatory components of the PI3K signaling pathway, the activator PIK3R3 and the inhibitor PTEN, in Ewing Sarcoma.

Materials and Methods

The human tissue sample research in this study was approved by the Colorado Multiple Institutional Review Board, which waived the need for specific donor or next of kin consent.

Cell lines and drugs

The cell lines A673, SK-ES-1, SK-N-MC, TC71 and EWS502, and corresponding culture conditions, have been described previously [12]. SK-PN-DW cells were obtained from ATCC. A4573 cells, described in [17], were obtained from Dr Timothy Triche. All cell lines were authenticated by STR profiling, performed at our institution, and determined to be mycoplasma-free. SK-PN-DW and A4573 cell lines were cultured in DMEM supplemented with 10% fetal bovine serum, Pen/Strep, 1 mM sodium pyruvate, 10 mM Hepes and 1X MEM nonessential amino acids. Drugs used were: actinomycin-D (Sigma, #A9415), doxorubicin (Sigma, #D1515), etoposide (CalBiochem, #341205), vincristine (Sigma, #V8879) and OSI-906 (Selleckchem, #S1091).

Protein expression analysis

Quantification of protein expression levels in cell lines was performed by immunoblotting, as previously described [12]. Primary antibodies used were: PIK3R3 (1:1000, Cell Signaling, #11889), PTEN (1:1000, Cell Signaling, #9552), pAkt (Ser473, 1:500, Cell Signaling, #4060L), Akt (1:1000, Cell Signaling, #9272), and tubulin (1:20000, Sigma, T5168). Secondary antibodies used were: goat anti-mouse IgG-HRP conjugate (1:5000, BioRad, #170-6516), goat anti-rabbit IgG-HRP conjugate (1:5000, BioRad, #170-6515).

Gene expression silencing

shRNA-mediated gene expression silencing via lentiviral delivery was performed as previously described [12]. The control, non-targeting shRNA consisted of a scrambled sequence (Addgene plasmid 1864; [18]). ShRNAs 1 and 2 for PIK3R3 correspond to TRCN0000195144 and TRCN0000197032 (Sigma Mission shRNA, distributed via the University of Colorado Cancer Center Functional Genomics Core Facility).

Ectopic protein expression

Full-length PTEN cDNA, with a C-terminal Myc/DKK epitope tag, was subcloned from pCMV6 (Origene) into the pCDH-Puro lentiviral expression vector (System Biosciences) using standard molecular techniques and fully sequence-verified. Preparation of replication-incompetent, VSV-G pseudotyped, infectious lentivirus, transduction of Ewing Sarcoma cells, and Puromycin selection (1 μg/mL) were performed as previously described [12].

Growth assays

Clonogenic and soft agar anchorage-independent growth assays were performed as previously described [19].

Drug sensitivity assays

Cells were plated in 96-well plates in triplicate. Plating cell numbers (optimized to ensure log-phase growth during the drug exposure) were: 2.25×10^4 for A673, A4573, SK-N-MC and EWS502 cells; 1.5×10^4 for SK-ES-1 and SK-PN-DW cells; and 3.75×10^3 for TC71 cells. 24 hours after plating, drug or vehicle control was added at the indicated concentration. The cells were then cultured for an additional 48 hours, at which point cell survival was measured using an MTT assay, as described [20]. IC50 values were determined via a non-linear regression plot performed using the GraphPad statistical software package.

Patient tumor samples and immunohistochemistry

For the analysis of PTEN expression in Ewing Sarcoma tumors, we adapted a recently published protocol for PTEN immunostaining in pathologic samples [21]. We first validated the staining protocol using our PTEN-positive and PTEN-negative cell lines, using the same specimen processing approach as described ([21] and as detailed below). We then stained formalin-fixed, paraffin-embedded tumor tissue specimens from 20 different patients, all verified as EWS/Ets fusion-positive Ewing Sarcomas during diagnostic work-up by the Children's Hospital Colorado (CHC) Pathology Department, using the identical protocol. For PTEN immunohistochemical staining, antigen was heat-retrieved in 10 mM Tris, 1 mM EDTA, pH 9.0, for 5 minutes at 125°C. PTEN antibody (Cell Signalling, #9559) was used at 1:100 dilution or 0.11 μg/mL; negative control primary antibody was rabbit IgG (Vector, #I-1000), also used at 0.11 μg/mL. Envision (anti-rabbit polymer, Dakocytomation) was used for detection. CD99 immunohistochemical staining was performed on a Ventana automated stainer, using an established diagnostic protocol in the CHC Pathology Department. CD99 antibody (Dakocytomation, #M3601) was used at 1:50 dilution; the negative control was mouse ascites fluid (Sigma, #M8273), used at 1:200 dilution. The iView DAB kit (Ventana) was used for detection. The studies were approved by our institutional IRB committee.

Results

The regulators of PI3K signaling, PIK3R3 and PTEN, are variably expressed in Ewing Sarcoma

Our laboratory had previously identified regulation of expression of components of the IGF signaling pathway by EWS/Fli1-controlled microRNAs in Ewing Sarcoma [12]. While evaluating candidate miR targets, we noted differential

expression of two regulators of the PI3K arm of tyrosine kinase signaling, PIK3R3 and PTEN. PIK3R3, a positive regulator of PI3K signaling, has recently been shown to be tumor-promoting in other cancers [22, 23], while PTEN, a negative regulator of this pathway, is an established tumor suppressor [24]. Examination of a panel of Ewing Sarcoma cell lines revealed approximately 3-fold variation in PIK3R3 protein levels (Fig. 1). Moreover, we noted loss of PTEN protein expression in one cell line (EWS502), which was associated with striking upregulation of baseline PI3K pathway activity (as determined by degree of AKT phosphorylation on serine 473; Fig. 1). These observations prompted us to undertake further studies of the potential roles of PIK3R3 and PTEN in the regulation of PI3K pathway activity and oncogenic phenotypes in Ewing Sarcoma.

PIK3R3 exerts growth-promotional effects in Ewing Sarcoma

To determine whether PI3KR3 plays a pro-oncogenic role in Ewing Sarcoma, as recently shown in other cancers [22, 23], we examined the effects of its depletion in Ewing Sarcoma cells. We selected SK-ES-1 cells for these experiments, as this cell line showed robust PIK3R3 expression and readily measurable baseline Akt phosphorylation (Fig. 1). We achieved stable depletion of PIK3R3 in SK-ES-1 cells using lentiviral delivery of two different targeting shRNAs (Fig. 2A). PIK3R3 depletion resulted in inhibition of growth in both a clonogenic assay (Fig. 2B, top panels) and a soft agar assay of anchorage-independent growth (Fig. 2B, bottom panels), in a manner that was proportional to the degree of PIK3R3 mRNA and protein knock-down (Fig. 2A). Interestingly, stable depletion of PIK3R3 had a variable effect on baseline Akt phosphorylation (Fig. 2C). Indeed, over multiple experiments, we did not observe a consistent decrease in the pAkt/Akt ratio in PIK3R3 depleted cells (Fig. 2C). Together, our findings indicate that PIK3R3 plays a growth-promotional role in Ewing Sarcoma, and suggest that this role is not strictly dependent on regulation of PI3K pathway activity. Of note, PI3K pathway-independent mechanisms have previously been reported for PIK3R3 in other cancers [25, 26].

PTEN expression is lost in a subset of Ewing Sarcoma cell lines and patient tumors

Our observation of the loss of PTEN, a known, potent, tumor suppressor, in a Ewing Sarcoma cell line (Fig. 1) was unexpected. We first sought to determine whether PTEN loss occurred in other Ewing Sarcoma cell lines. This analysis identified the additional cell lines A4573 and SK-PN-DW as also lacking PTEN protein expression (Fig. 3). As observed for EWS502 cells, both A4573 and SK-PN-DW cells also showed markedly higher levels of baseline Akt phosphorylation, in comparison to PTEN expressing lines (Fig 3). Next, we sought to verify that loss of PTEN expression occurs in patient tumors. To do this, we adapted a recently published protocol for PTEN immunostaining of formalin-fixed, paraffin-embedded tissue [21]. We first validated this protocol in our laboratory

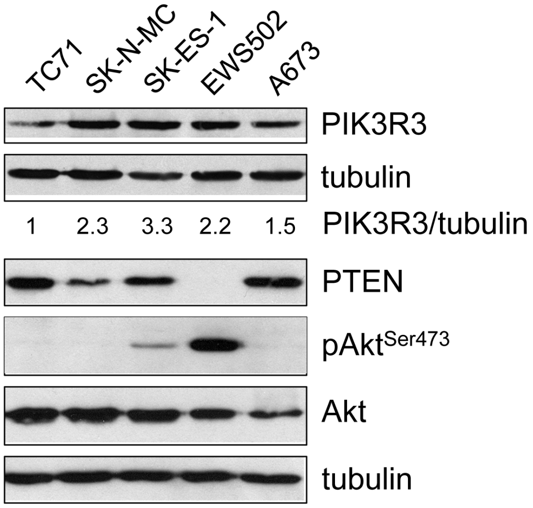

Fig. 1. Expression of PTEN and PIK3R3, and degree of Akt phosphorylation, in Ewing Sarcoma cell lines. The indicated Ewing Sarcoma cell lines (TC71, SK-N-MC, SK-ES-1, EWS502 and A673) were cultured in their respective media, and harvested at similar confluence (~70%) for protein extract preparation. Expression levels of PIK3R3, PTEN, pAkt (Ser473) and (total) Akt were determined using immunoblotting, with tubulin as the loading control; PIK3R3/tubulin ratios were determined by densitometry, with the ratio in TC71 cells arbitrarily set to 1.

doi:10.1371/journal.pone.0116895.g001

using our panel of PTEN-positive and PTEN-negative Ewing Sarcoma cell lines (S1 Fig.). We then examined PTEN expression in a panel of 20 EWS/Ets oncogenic fusion-verified patient tumor samples available at our institution (Children's Hospital Colorado). Nineteen of the twenty specimens demonstrated PTEN expression in tumor cells (the results for one such representative tumor are shown in Fig. 4, top panels; black arrowhead in the top right panel highlights a PTEN-positive tumor cell). One of the twenty tumors, however, demonstrated loss of PTEN expression in tumor cells (Fig. 4, bottom panels; note normal PTEN expression in vascular endothelium [white arrowhead, bottom right panel], but absence of PTEN expression in tumor cells [black arrow head, bottom right panel]. Thus, PTEN loss occurs in a subset of Ewing Sarcomas.

Effects of PTEN re-expression in Ewing Sarcoma cell lines lacking endogenous PTEN

To determine the functional consequences of loss of PTEN in Ewing Sarcoma, we stably re-introduced ectopic PTEN into two different PTEN-negative cell lines, EWS502 and A4573, using a lentiviral expression system (Fig. 5). The PTEN expression levels achieved were lower than those of endogenous PTEN in PTEN-positive lines (compare signal for A673 cells in the same blot; Fig. 5), presumably due to negative selection against high-level expression of a potent tumor suppressor. This partial PTEN re-expression was sufficient to enforce>50% downregulation of Akt phosphorylation in both cell lines (Fig. 5). Surprisingly, in spite of downregulation of PI3K pathway activity, PTEN re-expression failed to

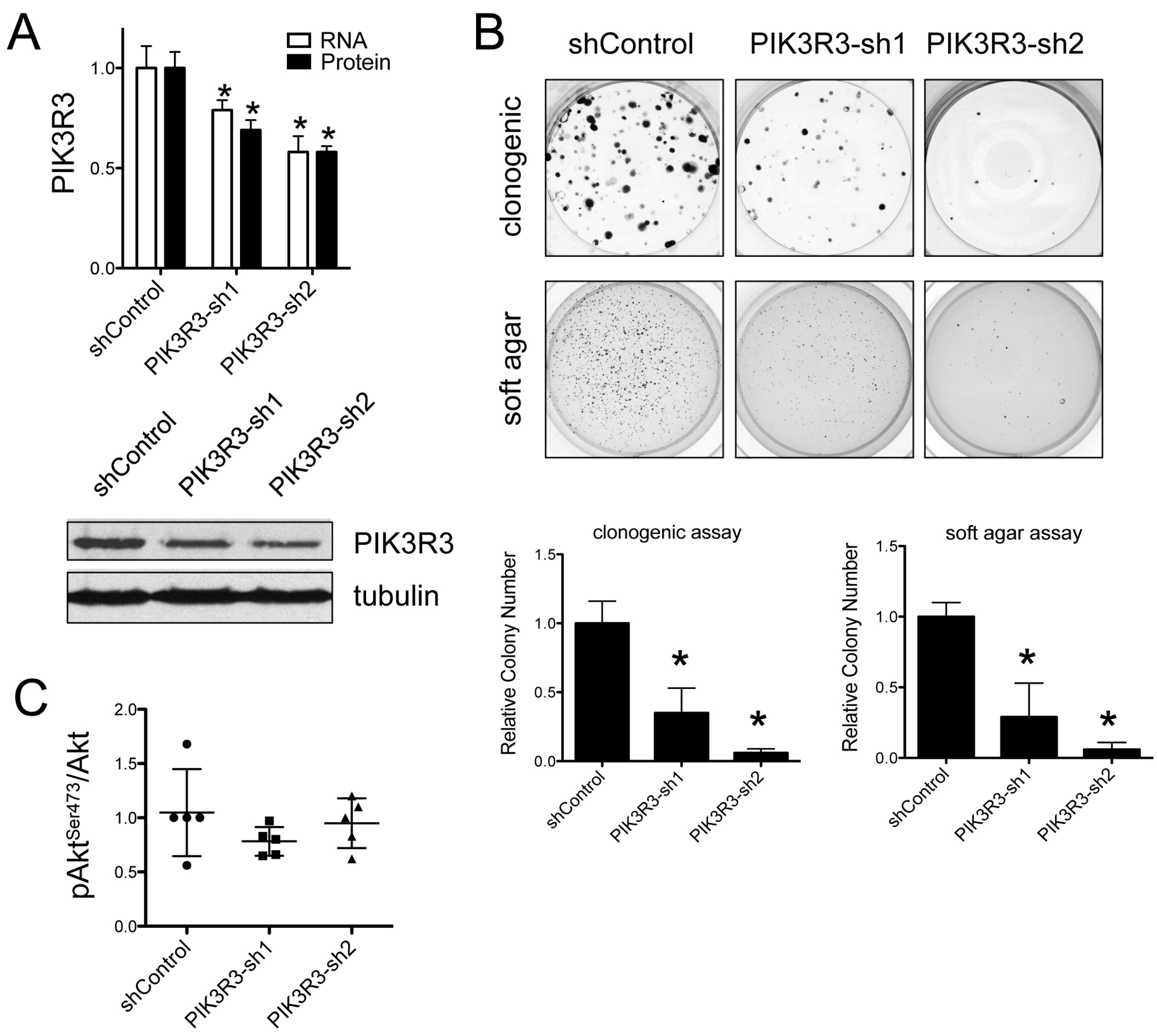

Fig. 2. PIK3R3 depletion results in inhibition of clonogenic and anchorage-independent growth of Ewing Sarcoma cells. (A) Stable depletion of PIK3R3 protein in Ewing Sarcoma SK-ES-1 cells was achieved using lentiviral delivery of two different targeting shRNAs (PIK3R3-sh1 and 2, described in Methods; shControl: cells expressing non-targeting scrambled sequence RNA, also described in Methods). PIK3R3 RNA levels were determined by qRT-PCR, as described previously [19], using specific primers to PIK3R3, and U6 as an endogenous control. PIK3R3 protein levels were determined by immunoblotting with PIK3R3 antibody, normalized to tubulin and quantified by densitometry. Mean and standard deviation are plotted at top, and a representative immunoblot is shown below. Mean values in the control groups are set to 1; *$p<0.05$, relative to corresponding control, using the student t-test. (B) Colony growth of control and PIK3R3-depleted SK-ES-1 cells as determined using a clonogenic assay (top panels and graph on left) and a soft agar assay of anchorage independent growth (bottom panels and graph on right). Representative images of individual wells from one experiment are shown. Quantifications represent the mean and standard error of the mean (SEM) of colony counts from independent experiments, each performed in triplicate (colony numbers in controls are set to 1; *$p<0.05$, relative to control, using the student t-test). (B) Levels of $pAkt^{Ser473}$ and total Akt were determined using immunoblotting as in Fig. 1, and quantified by densitometry. Plot shows relative $pAkt^{Ser473}$/Akt ratios from multiple experiments in control and PIK3R3-depleted SK-ES-1 cells; to facilitate comparison among different experiments, one value from control cells in each experiment is arbitrarily set to 1; bars show mean and standard deviation for each group. Statistical analyses did not reveal significant differences among the groups.

doi:10.1371/journal.pone.0116895.g002

inhibit growth in a clonogenic assay or a soft agar assay of anchorage-independent growth. In fact, partial PTEN re-expression resulted in a modest (~50%), but statistically significant, augmentation of anchorage-independent growth in soft agar (Fig. 6B; a smaller trend toward increased clonogenic growth was also seen in

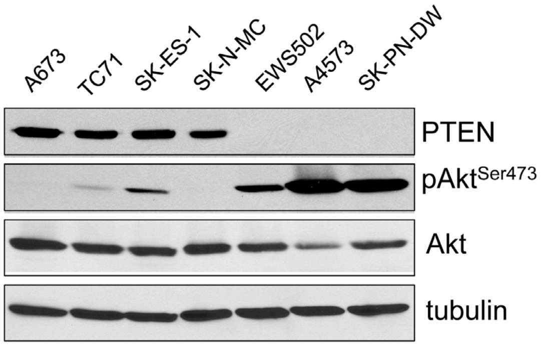

Fig. 3. PTEN-positive and PTEN-negative Ewing Sarcoma cell lines. The indicated cell lines were cultured in their respective media, and harvested at similar confluence (~70%) for protein extract preparation. Expression levels of PTEN, pAkt (Ser473) and (total) Akt were determined using immunoblotting, with tubulin as the loading control.

doi:10.1371/journal.pone.0116895.g003

the same experiments, but did not reach statistical significance [Fig. 6A]). While unexpected, this effect was observed in independent experiments, using de novo lentiviral transductions, in both cell lines.

Clonogenic and soft agar assays of anchorage-independent growth were performed as previously described [19]. Individual experiments were performed in triplicate, with 500 cells/well plated for clonogenic assays and 1×10^4 cells/well for soft agar assays. Experiments were independently repeated with de novo lentivirally transduced cells. Representative images of individual wells from one experiment are shown. Quantifications represent the mean and standard error of

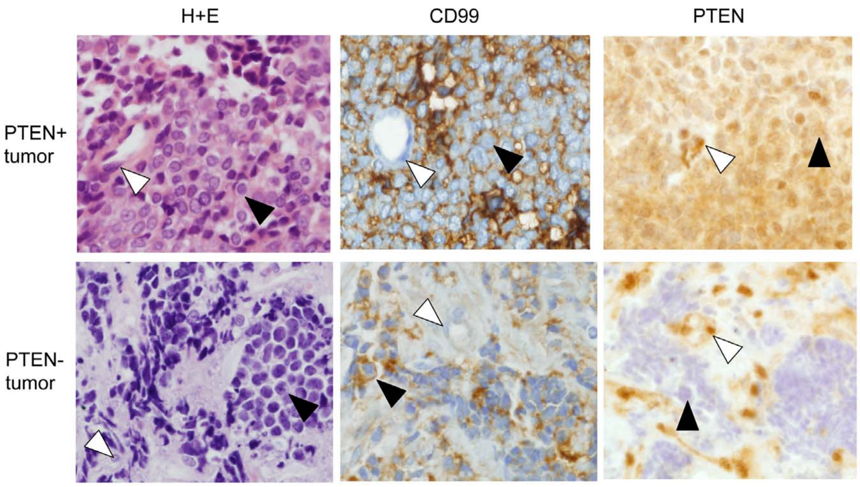

Fig. 4. PTEN expression in patient tumor samples. PTEN immunohistochemical staining in a representative PTEN-positive tumor and a PTEN-negative tumor. H+E histology and CD99 immunohistochemical staining are also shown. White arrowheads indicate representative vascular endothelial cells (strongly PTEN+ and CD99-); black arrowheads indicate representative tumor cells (CD99+). Note that vascular endothelial cells in both tumors show strong PTEN immunoreactivity of similar intensity (white arrowheads), but tumor cells only in the top tumor are PTEN immunoreactive (black arrowheads).

doi:10.1371/journal.pone.0116895.g004

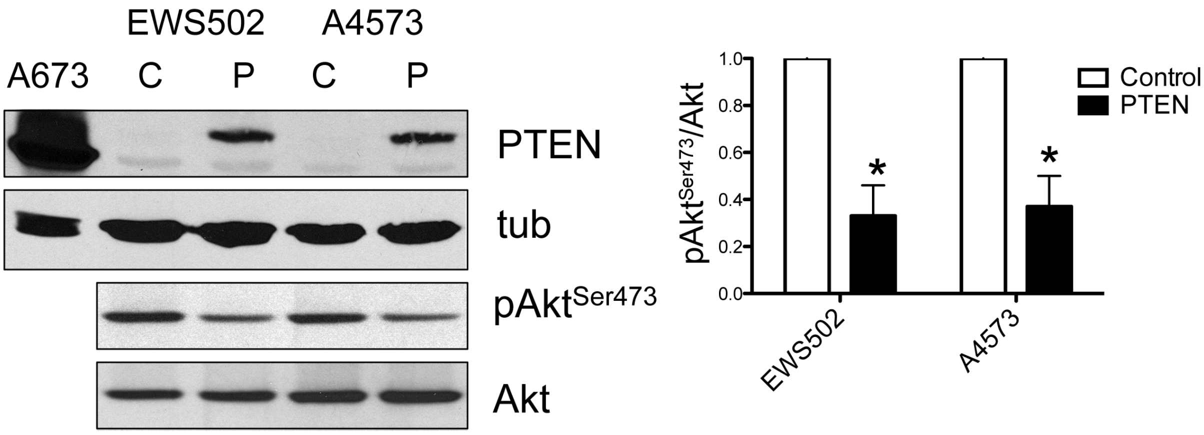

Fig. 5. Re-expression of PTEN in PTEN-negative Ewing Sarcoma cell lines. PTEN re-expression was achieved using a stable lentiviral expression system, and verified by immunoblotting with tubulin as loading control (C: control cells stably transduced with empty expression vector; P: cells stably transduced with PTEN expression construct; for reference, levels of endogenous PTEN in A673 cells are shown in the same blot). Consequences of PTEN re-expression on Akt phosphorylation (Ser473) are shown below, with quantification of $pAkt^{Ser473}$/Akt ratios from independent experiments shown on right (mean and standard deviation; values in control cells set to 1; *$p<0.05$, relative to control, using the student t-test).

doi:10.1371/journal.pone.0116895.g005

the mean (SEM) of colony counts from independent experiments, each performed in triplicate (colony numbers in controls are set to 1; *: $p<0.05$, using the student t-test).

We next examined the effects of PTEN status on drug response in Ewing Sarcoma. Re-expression of PTEN in PTEN-negative cells would be predicted to sensitize to inhibition of receptor tyrosine kinase action, by re-instating, at least partially, negative regulation of downstream signaling. To test whether this holds true in Ewing Sarcoma, we compared the efficacy of the IGF-1R small molecule inhibitor OSI-906 in PTEN-negative cells with and without ectopic PTEN expression. As shown in Fig. 7, we observed partial sensitization to OSI-906 upon PTEN re-expression in both cell lines, indicating that PTEN status contributes to sensitivity of Ewing Sarcoma cells to IGF-1R inhibition.

We additionally queried whether PTEN status affects the sensitivity of Ewing Sarcoma cells to conventional chemotherapeutic agents in use for therapy of this cancer, including actinomycin-D, etoposide, doxorubicin and vincristine. First, we asked whether drug response in our cell line panel would segregate by PTEN status. As shown in S2 Fig. and Fig. 8A, we observed such segregation for vincristine, but not the other agents. Namely, the PTEN-negative cell lines, EWS502, SK-PN-DW and A4573, demonstrated greater resistance to vincristine compared to the PTEN-positive lines. To determine whether PTEN indeed plays a causative role in this difference in sensitivity, we compared vincristine response in control and PTEN re-expressing EWS502 and A4572 cells. In both cell lines, re-introduction of PTEN resulted in a trend toward greater vincristine sensitivity. We conclude from these findings that PTEN levels may contribute to vincristine response in Ewing Sarcoma.

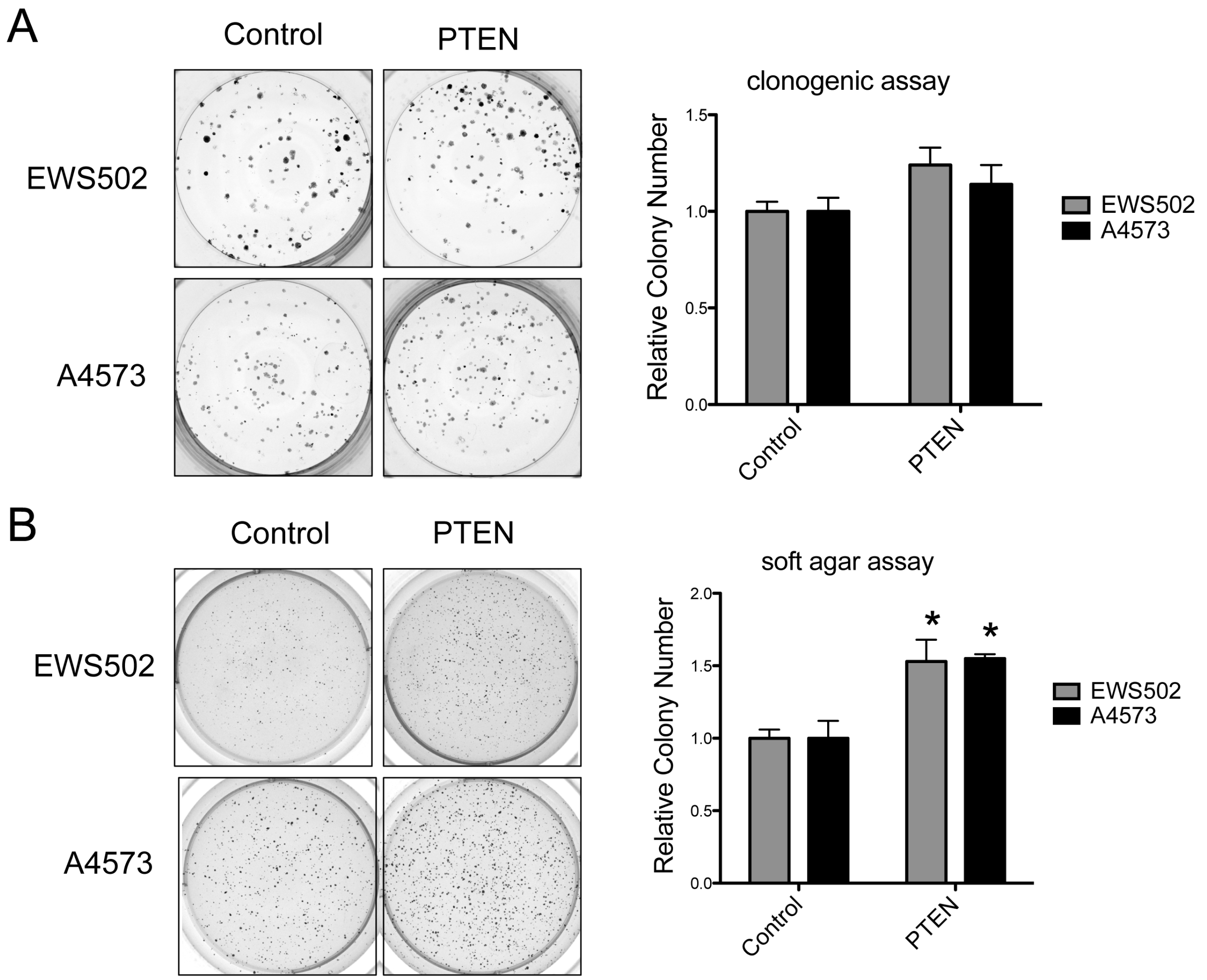

Fig. 6. Effects of PTEN re-expression on clonogenic and anchorage-independent growth of PTEN-negative Ewing Sarcoma cell lines.

doi:10.1371/journal.pone.0116895.g006

Discussion

Dysregulation of growth factor signaling pathways makes an important contribution to Ewing Sarcoma pathogenesis [2, 3], as true in many other cancers. However, in contrast to other cancers, the molecular basis of this dysregulation in Ewing Sarcoma is less well understood. Existing literature suggests that some degree of growth factor signaling pathway dysregulation can be attributed to downstream effects of the hallmark driver oncogenic fusion, the EWS/Ets chimeric oncoprotein or its variants [11–13]. However, studies also suggest that other, apparently EWS/Ets-independent, mechanisms also contribute [8–10]. Understanding such mechanisms is of importance as it may help shed light on the divergent biologic and clinical behavior of tumors bearing the same EWS/Ets oncogenic drivers. The PI3K signaling pathway illustrates this paradigm: it plays a very important role in Ewing Sarcoma biology [14, 15]; and it shows divergent activation across different cell lines bearing the same or similar fusions, the basis

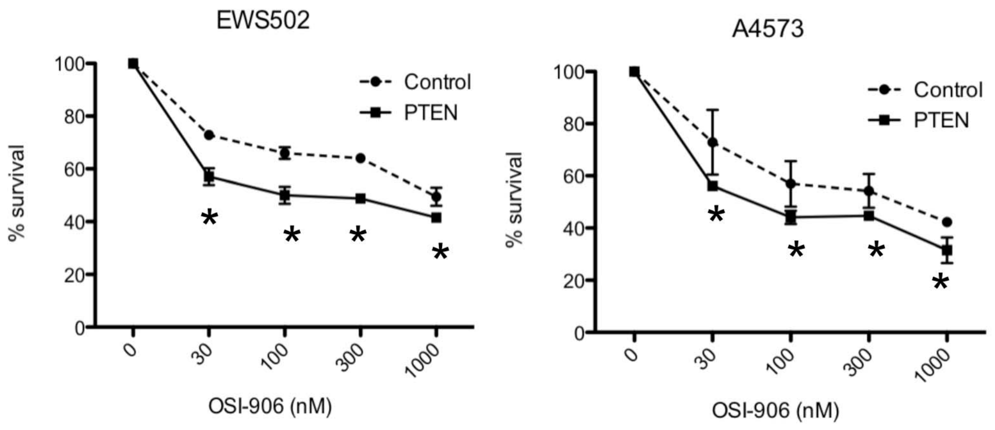

Fig. 7. Effects of PTEN re-expression on the response to IGF-1R blockade in PTEN-negative Ewing Sarcoma cell lines. Relative survival of control (dashed lines) and PTEN re-expressing (solid lines) cells in response to the IGF-1R small molecule inhibitor OSI-906 was determined using an MTT assay, as described in Methods. Results represent the mean and standard error of the mean of independent experiments, each performed in triplicate; $*p<0.05$, using the student t-test.

doi:10.1371/journal.pone.0116895.g007

of which is poorly understood [16]. In the present study, we asked how PIK3R3, a regulatory component of the PI3 kinase complex, and the phosphatase and established tumor suppressor PTEN, contribute to the control of PI3K pathway activity and oncogenic phenotypes in Ewing Sarcoma.

Our studies support a tumor-promotional role for PIK3R3 in Ewing Sarcoma. PIK3R3 has recently emerged as a tumor promoter in a number of malignancies, including glioblastoma [22], ovarian and colorectal carcinoma [23, 27], and leukemia [28]. Interestingly, these studies have identified multiple mechanisms that contribute to PIK3R3 tumor-promotional effects. Namely, in addition to its ability to enhance PI3K pathway activity [22], PIK3R3 has been shown to promote cell proliferation through interactions with the retinoblastoma protein (Rb) [25, 26], and to boost angiogenesis through the NF-κB pathway [27]. Our studies indicate that PIK3R3 growth-promotional effects in Ewing Sarcoma are not strictly dependent on regulation of PI3K pathway activity, and may operate through alternative mechanisms.

We further identify PTEN as a major determinant of PI3K pathway activation in Ewing Sarcoma. Namely, cell lines lacking PTEN show markedly higher baseline Akt phosphorylation, and reintroduction of PTEN, even at reduced levels compared to PTEN-positive cells, enforces robust downregulation of Akt phosphorylation. Consistent with prediction, our studies support a role for PTEN as a sensitizer to IGF receptor blockade, a biotherapy in clinical trials [29]. Interestingly, our studies also suggest that PTEN can modulate the sensitivity of Ewing Sarcoma cells to the microtubule inhibitor vincristine, a relevant conventional chemotherapeutic agent in this cancer. To our knowledge, effects of PTEN status on the response to microtubule inhibitors in cancer have not been reported. However, published reports have established a link between activated PI3K signaling via other mechanisms, and microtubule inhibitor resistance.

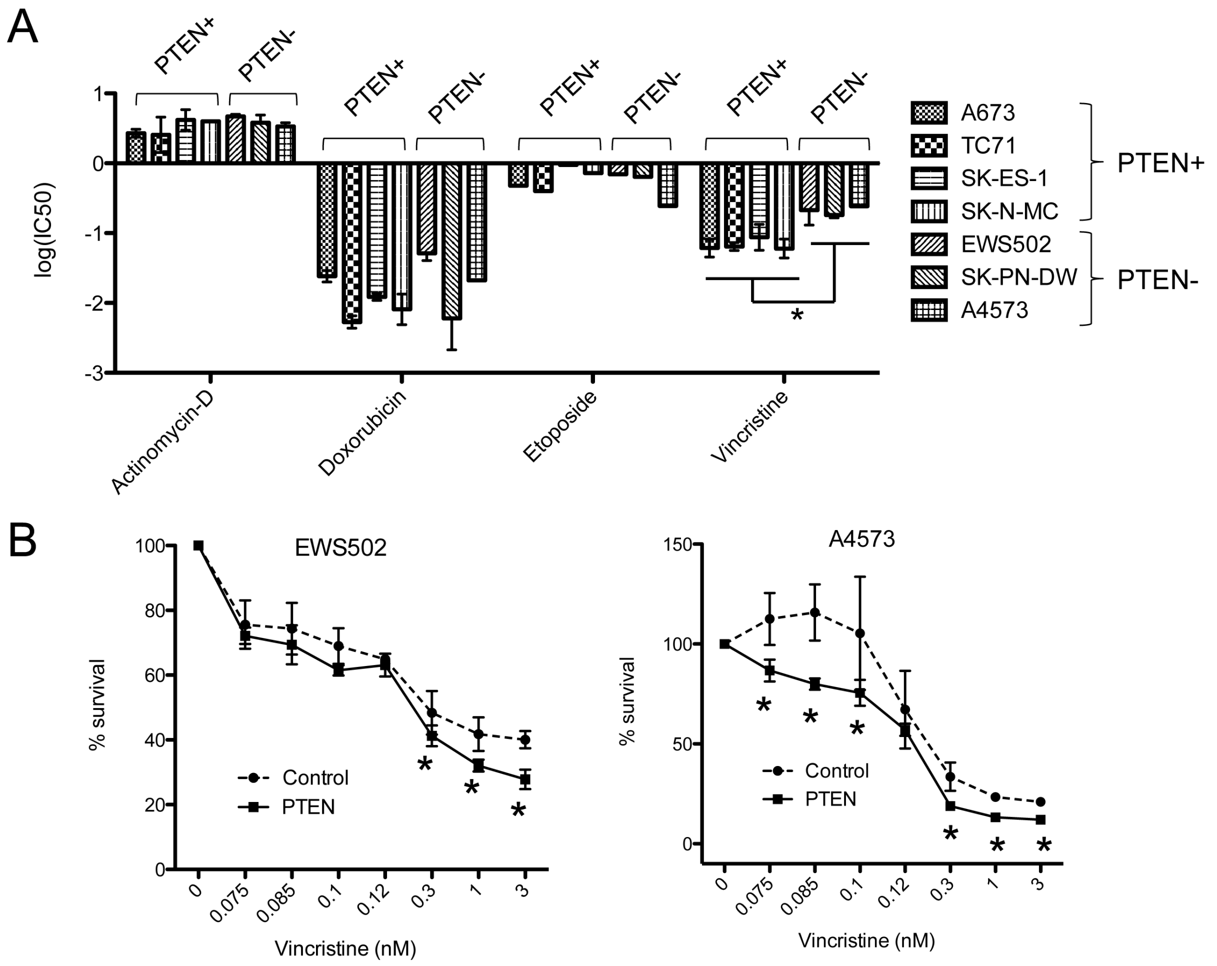

Fig. 8. PTEN expression and response of Ewing Sarcoma cells to conventional chemotherapeutic agents. (A) Cell survival upon treatment with the indicated agents was compared between PTEN-positive (PTEN+) and PTEN-negative (PTEN-) Ewing Sarcoma cell lines using an MTT assay (IC50 analysis based on data from S1 Fig.). IC50 values were determined using a non-linear regression plot. Data represent the mean and standard error of the mean of independent experiments, each performed in triplicate; *$p<0.05$, using the student t-test. (B) Relative survival of control (dashed lines) and PTEN-re-expressing (solid lines) cells in response to vincristine was determined using an MTT assay. Results represent the mean and standard error of the mean of independent experiments, each performed in triplicate; *$p<0.05$, using the student t-test.

doi:10.1371/journal.pone.0116895.g008

Specifically, constitutive Akt activity has been shown to promote resistance to microtubule inhibitors through activation of mTOR [30, 31], and induction of multidrug resistance pathways [32]. Similar mechanisms may contribute to the increased vincristine resistance observed in our studies of PTEN-negative Ewing Sarcoma cells.

A surprising observation in our studies was that reintroduction of PTEN into PTEN-negative Ewing Sarcoma cells resulted in modest augmentation of anchorage-independent growth, all in context of downregulation of Akt phosphorylation. The mechanistic basis of this unexpected finding is unclear. One possibility is that intermediate levels of PI3K pathway activity are favorable to growth, but at higher activity levels, this advantage is relinquished in favor of

augmented function of other cancer hallmarks, or, as suggested by our studies, increased drug resistance. Indeed, the cell state induced by partial PTEN restoration in our studies may be similar to PTEN haploinsufficiency, a state known to be able to support many oncogenic phenotypes, including increased cell proliferation [33]. It is also possible that, as in the case of PIK3R3, PI3K pathway-independent mechanisms play a role. A number of such mechanisms have now been described for PTEN, many involving nuclear functions [34–37], and it may be that some of these mechanisms have opposing (ie: promotional) effects on biological processes relevant to anchorage-independent growth in some contexts. Along similar lines, it is interesting to note that a recent report has identified transforming activity in a truncated form of PTEN [38]. We should also note that, in contrast to our findings, a recently published study by Patel, et al. observed inhibition of growth upon PTEN re-expression in Ewing Sarcoma EWS502 cells [39]. The difference in the findings of Patel, et al. and our studies may be explained by differences in PTEN expression levels and/or timing of phenotypic analysis (our studies, which used antibiotic selection prior to phenotypic analysis, may have selected for levels of PTEN re-expression permissive to cell growth).

In support of the disease relevance of PTEN loss, our studies identify absence of PTEN protein expression in one of twenty Ewing Sarcoma patient tumors in our institutional collection. While relatively low, this frequency is similar to the rate of p53 loss of function in Ewing Sarcoma (approximately one in ten tumors, across multiple studies) [40, 41]. Whether PTEN loss results in clinically more aggressive tumors, as suggested for p53 loss [40, 41], remains to be determined. A related question of interest is whether PTEN loss could represent a cooperating oncogenic event favoring Ewing Sarcoma initiation. Of note, since the present studies were undertaken, other reports have observed loss of PTEN in Ewing Sarcoma [39, 42, 43].

In summary, our studies identify a growth-promotional role for PIK3R3 in Ewing Sarcoma, and provide evidence that loss of PTEN expression may diminish responsiveness to vincristine and IGF-1R blockade. The latter observation may merit further investigation of PTEN as a potential predictive biomarker in Ewing Sarcoma therapy.

Supporting Information

S1 Fig. Validation of PTEN antibody for immunohistochemical staining. Specificity of PTEN immunohistochemical staining protocol was verified using formalin-fixed, paraffin-embedded pellets of PTEN-positive and PTEN-negative Ewing Sarcoma cell lines (as determined in Fig. 3). See Methods for protocol details.
doi:10.1371/journal.pone.0116895.s001 (TIF)

S2 Fig. Response of PTEN-positive and PTEN-negative Ewing Sarcoma cell lines to conventional chemotherapeutic agents. (A-D) Cell survival upon treatment with the indicated agents was compared between PTEN-positive (solid

lines) and PTEN-negative (dashed lines) using an MTT assay, as described in Methods.
doi:10.1371/journal.pone.0116895.s002 (TIF)

Acknowledgments

We wish to thank Timothy Triche for the A4573 cell line, Jonathan Fletcher for EWS502 cell line DNA for STR profiling, Rachel Linger and Deb DeRyckere for advice on chemotherapy survival assays, and Diana Cittelly for help with survival assay data analysis. We also wish to thank the University of Colorado Cancer Center DNA Sequencing, Flow Cytometry, Cell Culture and Functional Genomics Core Facilities, and the Barbara Davis Center Molecular Biology Core Facility.

Author Contributions

Conceived and designed the experiments: PJ. Performed the experiments: BFN JKP NSS TJ. Analyzed the data: BFN JKP NSS TJ JKR PJ. Wrote the paper: PJ.

References

1. **Ludwig JA** (2008) Ewing sarcoma: historical perspectives, current state-of-the-art, and opportunities for targeted therapy in the future. Curr Opin Oncol 20: 412–418.

2. **Jedlicka P** (2010) Ewing Sarcoma, an enigmatic malignancy of likely progenitor cell origin, driven by transcription factor oncogenic fusions. Int J Clin Exp Pathol 3: 338–347.

3. **Ordonez JL, Osuna D, Herrero D, de Alava E, Madoz-Gurpide J** (2009) Advances in Ewing's sarcoma research: where are we now and what lies ahead? Cancer Res 69: 7140–7150.

4. **Dylla L, Moore C, Jedlicka P** (2013) MicroRNAs in Ewing Sarcoma. Front Oncol 3: 65.

5. **Toomey EC, Schiffman JD, Lessnick SL** (2010) Recent advances in the molecular pathogenesis of Ewing's sarcoma. Oncogene 29: 4504–4516.

6. **Tsuchiya T, Sekine K, Hinohara S, Namiki T, Nobori T, et al.** (2000) Analysis of the p16INK4, p14ARF, p15, TP53, and MDM2 genes and their prognostic implications in osteosarcoma and Ewing sarcoma. Cancer Genet Cytogenet 120: 91–98.

7. **Olmos D, Tan DS, Jones RL, Judson IR** (2010) Biological rationale and current clinical experience with anti-insulin-like growth factor 1 receptor monoclonal antibodies in treating sarcoma: twenty years from the bench to the bedside. Cancer J 16: 183–194.

8. **Mendoza-Naranjo A, El-Naggar A, Wai DH, Mistry P, Lazic N, et al.** (2013) ERBB4 confers metastatic capacity in Ewing sarcoma. EMBO Mol Med 5: 1019–1034.

9. **Kamura S, Matsumoto Y, Fukushi JI, Fujiwara T, Iida K, et al.** (2010) Basic fibroblast growth factor in the bone microenvironment enhances cell motility and invasion of Ewing's sarcoma family of tumours by activating the FGFR1-PI3K-Rac1 pathway. Br J Cancer 103: 370–381.

10. **Sainz-Jaspeado M, Huertas-Martinez J, Lagares-Tena L, Martin Liberal J, Mateo-Lozano S, et al.** (2013) EphA2-induced angiogenesis in ewing sarcoma cells works through bFGF production and is dependent on caveolin-1. PLoS One 8: e71449.

11. **Herrero-Martin D, Osuna D, Ordonez JL, Sevillano V, Martins AS, et al.** (2009) Stable interference of EWS-FLI1 in an Ewing sarcoma cell line impairs IGF-1/IGF-1R signalling and reveals TOPK as a new target. Br J Cancer 101: 80–90.

12. **McKinsey EL, Parrish JK, Irwin AE, Niemeyer BF, Kern HB, et al.** (2011) A novel oncogenic mechanism in Ewing sarcoma involving IGF pathway targeting by EWS/Fli1-regulated microRNAs. Oncogene 30: 4910–4920.

13. **Prieur A, Tirode F, Cohen P, Delattre O** (2004) EWS/FLI-1 silencing and gene profiling of Ewing cells reveal downstream oncogenic pathways and a crucial role for repression of insulin-like growth factor binding protein 3. Mol Cell Biol 24: 7275–7283.

14. **Kang HG, Jenabi JM, Zhang J, Keshelava N, Shimada H, et al.** (2007) E-cadherin cell-cell adhesion in ewing tumor cells mediates suppression of anoikis through activation of the ErbB4 tyrosine kinase. Cancer Res 67: 3094–3105.

15. **Lawlor ER, Scheel C, Irving J, Sorensen PH** (2002) Anchorage-independent multi-cellular spheroids as an in vitro model of growth signaling in Ewing tumors. Oncogene 21: 307–318.

16. **Benini S, Manara MC, Cerisano V, Perdichizzi S, Strammiello R, et al.** (2004) Contribution of MEK/MAPK and PI3-K signaling pathway to the malignant behavior of Ewing's sarcoma cells: therapeutic prospects. Int J Cancer 108: 358–366.

17. **Navarro S, Gonzalez-Devesa M, Ferrandez-Izquierdo A, Triche TJ, Llombart-Bosch A** (1990) Scanning electron microscopic evidence for neural differentiation in Ewing's sarcoma cell lines. Virchows Arch A Pathol Anat Histopathol 416: 383–391.

18. **Sarbassov DD, Guertin DA, Ali SM, Sabatini DM** (2005) Phosphorylation and regulation of Akt/PKB by the rictor-mTOR complex. Science 307: 1098–1101.

19. **Parrish JK, Sechler M, Winn RA, Jedlicka P** (2013) The histone demethylase KDM3A is a microRNA-22-regulated tumor promoter in Ewing Sarcoma. Oncogene.

20. **Linger RM, DeRyckere D, Brandao L, Sawczyn KK, Jacobsen KM, et al.** (2009) Mer receptor tyrosine kinase is a novel therapeutic target in pediatric B-cell acute lymphoblastic leukemia. Blood 114: 2678–2687.

21. **Sangale Z, Prass C, Carlson A, Tikishvili E, Degrado J, et al.** (2011) A robust immunohistochemical assay for detecting PTEN expression in human tumors. Appl Immunohistochem Mol Morphol 19: 173–183.

22. **Soroceanu L, Kharbanda S, Chen R, Soriano RH, Aldape K, et al.** (2007) Identification of IGF2 signaling through phosphoinositide-3-kinase regulatory subunit 3 as a growth-promoting axis in glioblastoma. Proc Natl Acad Sci U S A 104: 3466–3471.

23. **Zhang L, Huang J, Yang N, Greshock J, Liang S, et al.** (2007) Integrative genomic analysis of phosphatidylinositol 3'-kinase family identifies PIK3R3 as a potential therapeutic target in epithelial ovarian cancer. Clin Cancer Res 13: 5314–5321.

24. **Yin Y, Shen WH** (2008) PTEN: a new guardian of the genome. Oncogene 27: 5443–5453.

25. **Hu J, Xia X, Cheng A, Wang G, Luo X, et al.** (2008) A peptide inhibitor derived from p55PIK phosphatidylinositol 3-kinase regulatory subunit: a novel cancer therapy. Mol Cancer Ther 7: 3719–3728.

26. **Xia X, Cheng A, Akinmade D, Hamburger AW** (2003) The N-terminal 24 amino acids of the p55 gamma regulatory subunit of phosphoinositide 3-kinase binds Rb and induces cell cycle arrest. Mol Cell Biol 23: 1717–1725.

27. **Wang G, Chen C, Yang R, Cao X, Lai S, et al.** (2013) p55PIK-PI3K stimulates angiogenesis in colorectal cancer cell by activating NF-kappaB pathway. Angiogenesis 16: 561–573.

28. **Wang G, Deng Y, Cao X, Lai S, Tong Y, et al.** (2012) Blocking p55PIK signaling inhibits proliferation and induces differentiation of leukemia cells. Cell Death Differ 19: 1870–1879.

29. **Grohar PJ, Helman LJ** (2013) Prospects and challenges for the development of new therapies for Ewing sarcoma. Pharmacol Ther 137: 216–224.

30. **Vanderweele DJ, Rudin CM** (2005) Mammalian target of rapamycin promotes vincristine resistance through multiple mechanisms independent of maintained glycolytic rate. Mol Cancer Res 3: 635–644.

31. **VanderWeele DJ, Zhou R, Rudin CM** (2004) Akt up-regulation increases resistance to microtubule-directed chemotherapeutic agents through mammalian target of rapamycin. Mol Cancer Ther 3: 1605–1613.

32. **Zhang H, Li B, Bai SW, Wang HJ** (2010) Constitutively active Akt contributes to vincristine resistance in human retinoblastoma cells. Cancer Invest 28: 156–165.

33. **Trotman LC, Niki M, Dotan ZA, Koutcher JA, Di Cristofano A, et al.** (2003) Pten dose dictates cancer progression in the prostate. PLoS Biol 1: E59.

34. **Li AG, Piluso LG, Cai X, Wei G, Sellers WR, et al.** (2006) Mechanistic insights into maintenance of high p53 acetylation by PTEN. Mol Cell 23: 575–587.

35. **Shen WH, Balajee AS, Wang J, Wu H, Eng C, et al.** (2007) Essential role for nuclear PTEN in maintaining chromosomal integrity. Cell 128: 157–170.

36. **Song MS, Carracedo A, Salmena L, Song SJ, Egia A, et al.** (2011) Nuclear PTEN regulates the APC-CDH1 tumor-suppressive complex in a phosphatase-independent manner. Cell 144: 187–199.

37. **Chen ZH, Zhu M, Yang J, Liang H, He J, et al.** (2014) PTEN Interacts with Histone H1 and Controls Chromatin Condensation. Cell Rep 8: 2003–2014.

38. **Fernandez S, Genis L, Torres-Aleman I** (2014) A phosphatase-independent gain-of-function mutation in PTEN triggers aberrant cell growth in astrocytes through an autocrine IGF-1 loop. Oncogene 33: 4114–4122.

39. **Patel M, Gomez NC, McFadden AW, Moats-Staats BM, Wu S, et al.** (2014) PTEN Deficiency Mediates a Reciprocal Response to IGFI and mTOR Inhibition. Mol Cancer Res 12: 1610–1620.

40. **Neilsen PM, Pishas KI, Callen DF, Thomas DM** (2011) Targeting the p53 Pathway in Ewing Sarcoma. Sarcoma 2011: 746939.

41. **Shukla N, Schiffman J, Reed D, Davis IJ, Womer RB, et al.** (2013) Biomarkers in Ewing Sarcoma: The Promise and Challenge of Personalized Medicine. A Report from the Children's Oncology Group. Front Oncol 3: 141.

42. **Lynn M, Wang Y, Slater J, Shah N, Conroy J, et al.** (2013) High-resolution genome-wide copy-number analyses identify localized copy-number alterations in Ewing sarcoma. Diagn Mol Pathol 22: 76–84.

43. **Manara MC, Nicoletti G, Zambelli D, Ventura S, Guerzoni C, et al.** (2010) NVP-BEZ235 as a new therapeutic option for sarcomas. Clin Cancer Res 16: 530–540.
